# Gas-phase detection of solid-state fission product complexes for post-detonation nuclear forensic analysis

**DOI:** 10.1007/s10967-016-4920-4

**Published:** 2016-06-27

**Authors:** S. Adam Stratz, Steven A. Jones, Colton J. Oldham, Austin D. Mullen, Ashlyn V. Jones, John D. Auxier, Howard L. Hall

**Affiliations:** 1 Department of Nuclear Engineering, University of Tennesse, 315 Pasqua Engineering Bldg, Knoxville, TN 37996 USA; 2 Radiochemistry Center of Excellence, University of Tennessee, 1508 Middle Dr., Knoxville, TN 37996 USA; 3 Bredeson Center for Interdisciplinary Research, University of Tennessee, 1640 Cumberland Ave., Knoxville, TN 37996 USA; 4 Department of Chemistry, University of Tennessee, 552 Buehler Hall, 1420 Circle Dr., Knoxville, TN 37996 USA; 5 Institute for Nuclear Security, University of Tennessee, 1640 Cumberland Ave., Knoxville, TN 37996 USA

**Keywords:** Nuclear forensics, Rare earth separations, Nuclear security, Post-detonation

## Abstract

This study presents the first known detection of fission products commonly found in post-detonation nuclear debris samples using solid sample introduction and a uniquely coupled gas chromatography inductively-coupled plasma time-of-flight mass spectrometer. Rare earth oxides were chemically altered to incorporate a ligand that enhances the volatility of the samples. These samples were injected (as solids) into the aforementioned instrument and detected for the first time. Repeatable results indicate the validity of the methodology, and this capability, when refined, will prove to be a valuable asset for rapid post-detonation nuclear forensic analysis.

## Introduction

Debris generated in a nuclear detonation is of special interest to the nuclear forensics community. Components of the weapon along with fission products, activation products, and material from the surrounding environment are swept into the fireball and can condense into a glassy matrix known as nuclear melt glass [1]. The melt glass encapsulates these constituents and gives the forensic scientist an indication of the chemical, radiological, and neutronic conditions at the epicenter of the blast. Such detailed information, if identified and quantified from the melt glass, is crucial for the technical nuclear forensic component of the attribution cycle. This information is combined with intelligence and law enforcement details to make an informed attribution decision, and it is crucial that the scientific analysis of the debris fed into this cycle is performed with both speed and precision. Current methodologies to separate and quantify nuclear melt glass constituents, including precipitation, solvent extraction, etc., require lengthy analytical techniques that can take several hours to perform [2, 3]; this research aims to reduce the time needed for technical analysis to minutes, or even seconds, by exploiting gas-phase chemistry and a solid injection methodology [4, 5]. Results have indicated for the first time that appropriate instrumentation has been developed to detect fission product complexes using solid-state injection.

## Theory

Rare earth (RE) elements comprise the heavy end of the plutonium and uranium fission product spectrums and require quantification in a post-detonation scenario. These elements frequently demonstrate isobaric interferences with one another, and with the exception of Ce, Tb, and Eu, are trivalent and exhibit coordination numbers ranging from 8 to 9. In traditional nuclear forensic analysis, the rare earths are often removed from actinides of interest using column chromatography chemistry to remove fission products and trace elements prior to aliquoting the samples for various measurement techniques. Mass spectrometry detection measures a mass to charge ratio and typically differentiates between mass numbers, not elements themselves [6]. To reduce analysis time, we have explored the feasibility of performing RE separations in the gas phase to expedite the analytical process. A gas chromatography (GC) instrument was attached to an inductively-coupled plasma time-of-flight mass spectrometer (ICP-TOF–MS) in order to first separate the fission products based on their gaseous thermodynamic properties, then detect individual subsequent mass number elution after complete obliteration of the molecule in the ICP-TOF-MS system. This allows for isobaric interferences to be significantly reduced when quantifying the elemental and isotopic RE composition of the sample.

## Experimental

RE fission products, denoted “Ln”, are often found in their oxide state (e.g., Ln_2_O_3_) in nuclear melt glass [7]. However, oxides are intrinsically non-volatile at traditional GC operating temperatures. For this research, the oxides were made volatile using a ligand known as 1,1,1,5,5,5-hexafluoro-2,4-pentanedione (hereafter referred to as hfac). This NH_4_Ln(hfac)_4_ compound exhibits sublimation temperatures across the Ln series ranging from 140 to 250 °C, which can be attained by a conventional GC instrument. The NH_4_Ln(hfac)_4_ complexes were synthesized using RE oxides (Sigma Aldrich, 99.99 %) and combined with concentrated HCl (Fisher, ACS Reagent Grade) to form a chloride complex. Hfac (Acros, 99.9 %) was obtained and combined with equimolar amounts of concentrated NH_4_OH (Fisher, ACS Reagent Grade) at 0 °C. The two liquids reacted to produce a solid (NH_4_[hfac]) that was subsequently stirred to ensure full reaction and desiccated. A small amount of water, approximately 50 µL, was used to dissolve the chloride complex. The NH_4_[hfac] was dissolved in 5 mL of diethyl ether (ACS Reagent Grade, Fisher) and added to the RE chloride solution in a 4:1 ratio. The mixture was strongly shaken for 30 s, set for 5 min, and repeated three times. After the last agitation, the organic phase was drawn off and desiccated to dry the sample and remove the ether. The entire process requires approximately 90 min.

**Fig. 1:**
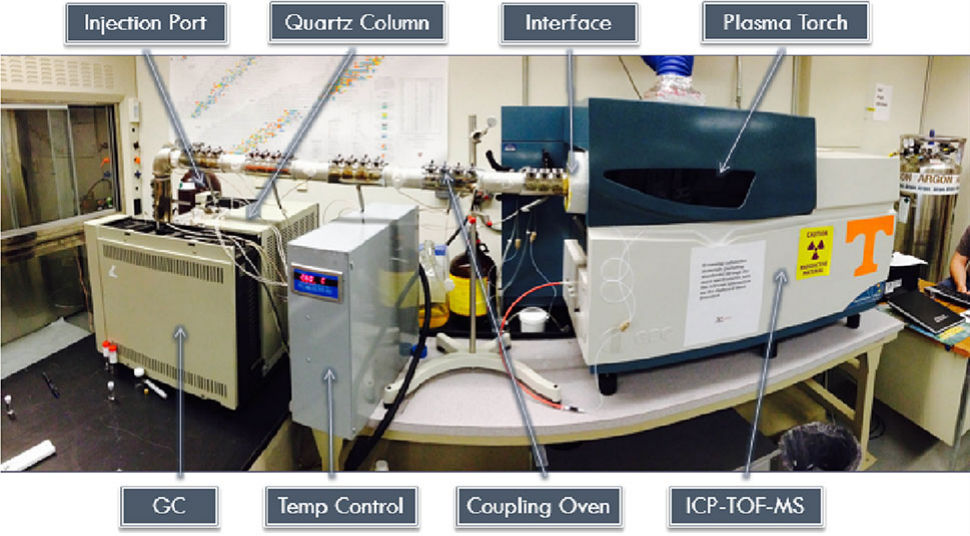
GC-ICP-TOF-MS setup

**Fig. 2:**
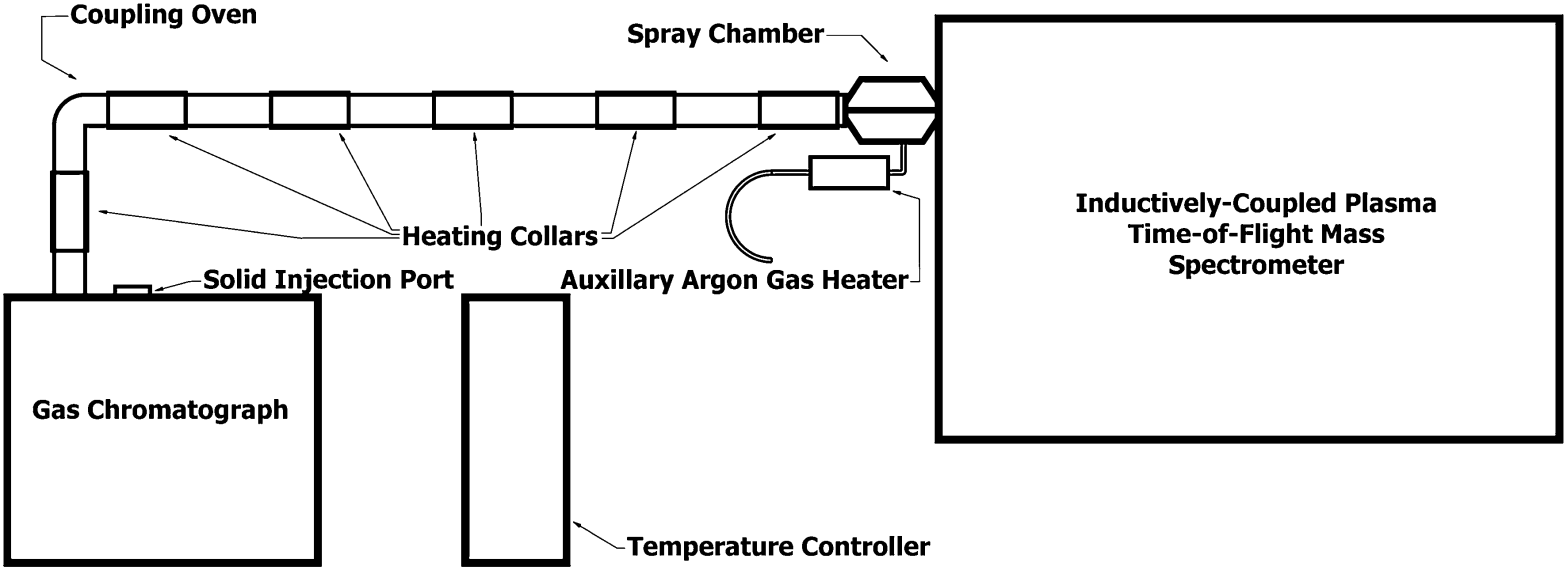
Schematic of GC-ICP-TOF-MS setup

The resulting solid RE hfac complexes were ready for direct injection into a GC retrofitted to allow for a large-bore syringe. The present article presents results from a NH_4_Pr(hfac)_4_ injection. A minute amount of the Pr complex was packed into the needle end of a disposable syringe with the plunger fully drawn. The sample was injected into the coupled GC-ICP-TOF-MS instrument, which is shown in Fig. 1 and schematically represented in Fig. 2. The GC is a Hewlett-Packard 5890A set to 200 °C with argon carrier gas flowing at approximately 7 mL/min through a 7-m uncoated 0.53 mm I.D. Agilent quartz column. Seven individual thermocouples are attached to a control box to heat a 1.5″ I.D. Stainless steel tube that connects the GC to the ICP-TOF–MS.

The temperature programming unit is an Omega CN1504 multi-zone controller with four heating control zones. The nebulizer flow carrying the sample from the end of the quartz column to the plasma torch in the ICP-TOF-MS was also heated using one of the four control zones and a custom quartz heating coil. Mass spectra were recorded using a GBC Optimass 9500 ICP-TOF-MS with 1200 W plasma power and a 0.950 L/min nebulizer flow.

## Results

**Fig. 3:**
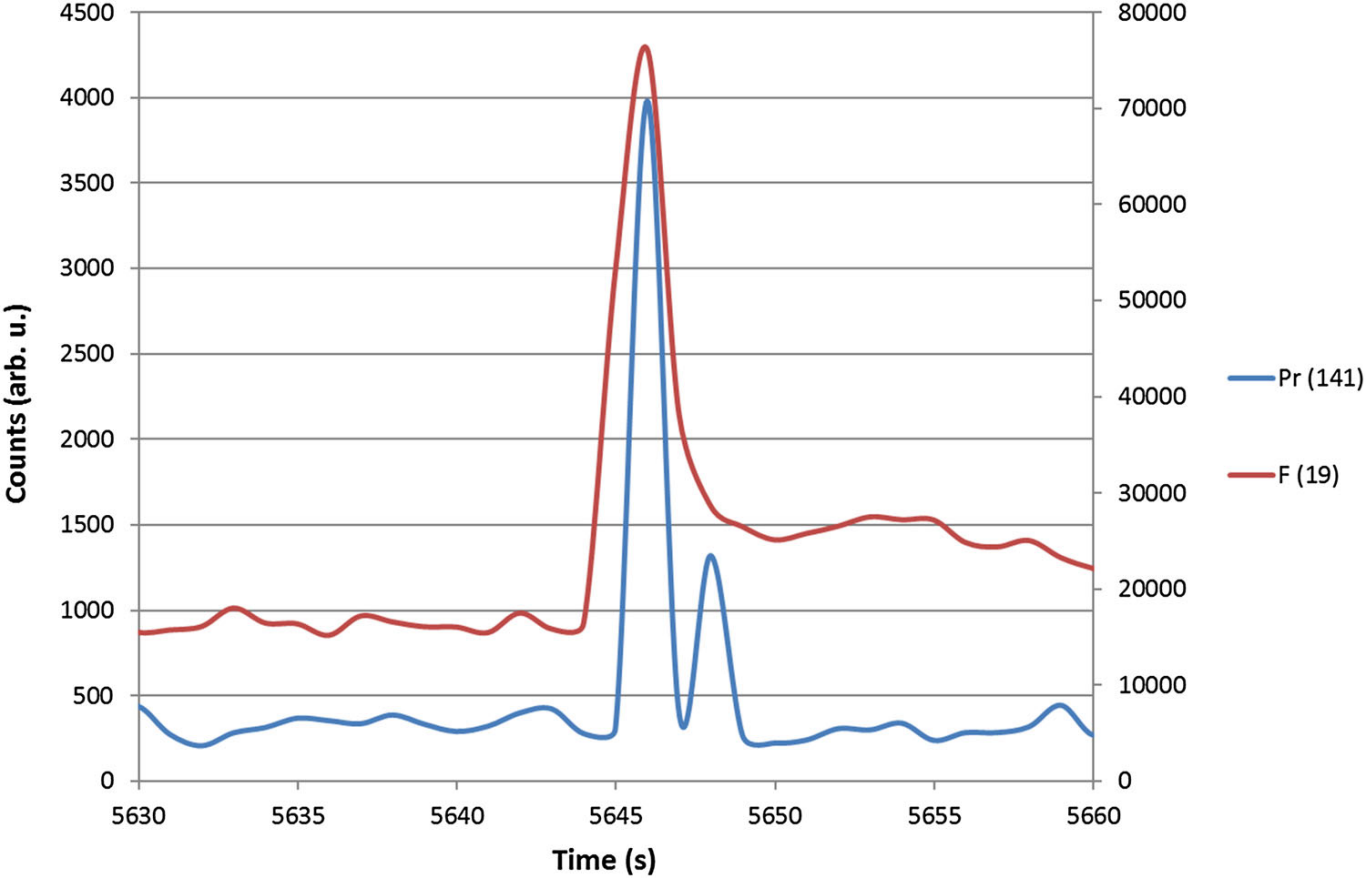
Pr and F detection

The solid NH_4_Pr(hfac)_4_ sample was injected into the inlet port on the GC and was detected after 28 s. Figure 3 shows the Pr peak along with the associated F peak resulting from the attached fluorinated ligands. It should be noted that the ICP ionization system removes the ability to determine molecular peaks, and the working range of the MS system is 19–310 amu.

The simultaneous fluorine peak indicates that the NH_4_Pr(hfac)_4_ complex was fully intact after traveling through the column and arriving at the plasma torch. It is extremely important that the molecule stay intact throughout the process due to the highly non-volatile nature of Pr (and the other RE metals). If any Pr is separated from its associated ligands, it will precipitate onto the quartz column and irreversibly adsorb.

Ensuring the whole injected sample elutes is the only reliable means of providing accurate quantitative results from this system in future experiments. However, the injection shown in Fig. 3 should only be treated qualitatively and demonstrates that the injected sample successfully and quickly traversed the entire setup; therefore, no quantitative error analysis was performed as we were simply attempting to determine both elution feasibility and instrument continuity during preliminary trial runs. Successful peak elution, particularly of both the fission product and associated ligand eluting simultaneously, confirms that the chemistry and volatility of the samples will perform successfully in this experimental setup. From this experiment, we have confirmed that gas-phase chemistry can be successfully exploited to detect fission product samples for post-detonation nuclear forensic analysis. Quantitative experiments to investigate elution yield are the next step of this process, and separations using multiple fission product complexes will follow soon thereafter.

## Conclusions

Results indicate the successful development of the first known instrumentation and methodology to identify and quantify RE fission product complexes that would occur in nuclear melt glass using gas-phase chemistry with direct solid sample injection. The detection was rapid, occurring only 28 s after the sample was initially injected. Work is currently underway to further reduce total sample analysis time by modifying the apparatus with a high-temperature furnace for direct injection of chlorides (and eventually oxides) for separation and detection without chemical alteration. Many issues need resolving before a transition to non-volatile sample injection can occur, but even considering the current chemical synthesis step, a sample can be analyzed from its organometallic state in significantly less time, on the order of seconds to minutes, when compared to current solvent extraction or precipitation techniques that can take several hours to perform [3].
